# Influence of Sex and a High-Fiber Diet on the Gut Microbiome of Alentejano Pigs Raised to Heavy Weights

**DOI:** 10.3390/vetsci10110641

**Published:** 2023-11-02

**Authors:** André Albuquerque, Nicolás Garrido, Rui Charneca, Conceição Egas, Luísa Martin, Amélia Ramos, Filipa Costa, Carla Marmelo, José Manuel Martins

**Affiliations:** 1 ECO-PIG Consortium, Z.I. Catraia, Ap. 50, 3440-131 Santa Comba Dão, Portugal; nicolas.osa@uevora.pt (N.G.); rmcc@uevora.pt (R.C.); luisam@esac.pt (L.M.); ameliaramos@esac.pt (A.R.); lipaaa.costa@gmail.com (F.C.); carla.marmelo@racoessantiago.pt (C.M.); 2 MED—Mediterranean Institute for Agriculture, Environment and Development & CHANGE—Global Change and Sustainability Institute, Universidade de Évora, Pólo da Mitra, Ap. 94, 7006-554 Évora, Portugal; 3 Escola Superior Agrária de Elvas, Departamento de Ciência Agrárias e Veterinárias, Edifício Quartel do Trem, Avenida 14 de Janeiro n° 21, 7350-092 Elvas, Portugal; 4 MED & CHANGE, Departamento de Zootecnia, ECT–Escola de Ciências e Tecnologia, Universidade de Évora, Pólo da Mitra, Ap. 94, 7006-554 Évora, Portugal; 5 Center for Neuroscience and Cell Biology, University of Coimbra, 3004-504 Coimbra, Portugal; conceicao.egas@biocantassociacao.pt; 6 Next Generation Sequencing Unit, Biocant, 3060-197 Cantanhede, Portugal; 7 Departamento de Ciências Agrárias e Tecnologias, Escola Superior Agrária de Coimbra, Bencanta, 3045-601 Coimbra, Portugal

**Keywords:** gut microbiota, fiber rich diets, castration, local Portuguese pig breed, complex carbohydrate fermentation bacteria, outdoor rearing

## Abstract

**Simple Summary:**

The concept of modernization of pig production systems should not rely only on new technological inputs and opportunities, but should also address societal expectations regarding animal well-being while maintaining product quality. The Alentejano (AL) pig is a fatty breed from Portugal traditionally raised outdoors, surgically castrated, and slaughtered at heavy weights for its renowned high-quality meat and meat products. The gastrointestinal tract of pigs harbors a diverse ecosystem of microorganisms that play a major role in feed digestion, nutrient absorption, and in the synthesis and metabolism of skatole, one of the main sources of the unpleasant boar taint. Our study aimed to investigate gut microbiota changes in heavy AL pigs fed with a new high-fiber diet, aiming to reduce the skatole-producing microbiota, while also exploring the effects of surgical castration in that community. The pigs that were fed the new diet presented higher abundances of species responsible for breaking down complex carbohydrate substrates, while castrated pigs presented lower abundances of probiotic bacteria, normally associated with better gut health and lower body fat. Utilizing fiber-enriched diets remains a promising approach to address boar taint in intact animals, especially when sourced from locally produced legumes and agricultural by-products, which can also contribute to broader sustainability objectives.

**Abstract:**

This study investigates the influence of sex and a dietary transition on the gut microbiota of a local Portuguese pig breed. Three groups of male Alentejano pigs (*n* = 10 each) were raised between ~40 and 160 kg LW. Group C included pigs that were surgically castrated, while the I group included intact ones; both were fed with commercial diets. The third group, IExp, included intact pigs that were fed commercial diets until ~130 kg, then replaced by an experimental diet based on legumes and agro-industrial by-products between ~130 and 160 kg. Fecal samples were collected two weeks before slaughter. The total DNA was extracted and used for 16S metabarcoding on a MiSeq^®^ System. The dietary transition from a commercial diet to the experimental diet substantially increased and shifted the diversity observed. Complex carbohydrate fermenting bacteria, such as *Ruminococcus* spp. and *Sphaerochaeta* spp., were significantly more abundant in IExp (q < 0.05). On the other hand, castrated pigs presented a significantly lower abundance of the potential probiotic, *Roseburia* spp. and *Lachnospiraceae* NK4A136 group (q < 0.01), bacteria commonly associated with better gut health and lower body fat composition. Understanding the role of gut microbiota is paramount to ensure a low skatole deposition and consumers’ acceptance of pork products from non-castrated male pigs.

## 1. Introduction

One of the main contemporary welfare concerns regarding pig production resides in the controversial decision to surgically castrate male pigs. This traditionally adopted practice is used to minimize aggressive behaviors and the occurrence of undesired boar taint in the meat and fat of these animals [1,2]. Boar taint in intact (non-castrated or entire) animals is characterized by the potential production of an unpleasant odor and flavor, which is rejected by consumers when detected. This issue is mostly attributed to the overaccumulation in adipose tissue of androstenone (5-androst-16-ene-3-one), skatole (3-methyllindole), and, to a lesser extent, indole. The deposition of these compounds in carcasses is a remarkably intricate process influenced by a multitude of factors including age, hormone balance, feeding, microbial activity, and genetic background, among others [3,4,5,6].

Androstenone is a steroid synthesized by Leydig cells in the testes and is strongly linked with sexual development. The production of this hormone is known to increase with age, and its tissue deposition causes an unpleasant urinous scent and flavor in cooked meat, with acceptable levels for consumers ranging from 500 to 1000 ng/g of fat [3,7].

Skatole is a metabolite produced during the decomposition of the amino acid, L-tryptophan, by microbes in the digestive tract of mammals [7,8]. Like androstenone, skatole is also known to increase with age, particularly since its degradation process can be inhibited by the presence of androstenone [5]. Deposited skatole in fat tissues is responsible for its feculent scent, and acceptable levels for consumers generally range from 200 to 250 ng/g of fat [7].

With public pressure requiring European stakeholders to sign a declaration to end surgical castration, it is necessary to find solutions to overcome the potential drawbacks of raising intact pigs, particularly the occurrence of boar taint [5]. One way to achieve this is through diet manipulation, although the involved mechanisms are often uncertain and the results occasionally inconsistent [6]. Chicory roots can reduce the amount of skatole deposited by increasing the activity of the cytochrome P450 complex [9]. They also have been suggested to reduce androstenone accumulation by stimulating 3β-hydroxysteroid dehydrogenase [10]. Diets rich in fiber provide new substrates for the gut microbiome and promote the growth of anaerobic bacteria that break down carbohydrates, instead of protein, to obtain energy. Fermentation of these carbohydrates (pectin, hemicellulose, lignin, inulin, and resistant starch) result in the production of short-chain fatty acids, such as acetate, propionate, and butyrate, which have been shown to positively influence gut health and reduce the deposition of boar taint compounds [11,12,13].

The gastrointestinal tract of pigs harbors a diverse and dynamic ecosystem of microorganisms that have established a close symbiotic relationship with their host. These can play a major role in feed digestion and nutrient absorption, prevent pathogen colonization, and contribute to the maturation and education of the immune system [14,15]. Gut bacteria are fundamental in breaking down cellulose and other complex carbohydrates since animals cannot digest them, and, in pigs, hindgut bacterial fermentation in the colon can account for up to 25% of the total energy produced [13,16]. The Alentejano (AL) pig is a breed traditionally raised outdoors, surgically castrated, and slaughtered at heavy weights for its distinguished high-quality meat and meat products. This breed is characterized by low growth rates, which contrast with the elevated and precocious ability to accumulate and store fat [17,18,19]. AL pigs are mostly bred in the southern region of Portugal and are known to be genetically similar to the Iberian breed [20,21]. Renowned for their robustness and adaptability, these pigs thrive under extensive production systems, taking advantage of the grass and acorns available in the “montado” ecosystem, blending with the region’s natural landscape and with the local cultural heritage [17,22].

The potential impacts of surgical castration and dietary transitions in the gut microbiome are underexplored fields of research, particularly in local pig breeds being raised to heavy weights. For the certification of some dry-cured products from this breed, like the high-grade hams, the animals can only be slaughtered at an age of at least 14 months and a bodyweight ranging from 145 to 210 kg since the quality of the products is related to the age, weight, and diet of the animals [23,24]. Most similar studies use modern genotypes that are generally less adapted to digest dietary fiber due to historic breeding goals focused on increasing production and performance traits. This may have decreased the ability of these pigs to break down complex carbohydrates and make an effective use of dietary fiber. In contrast, autochthonous breeds, such as the AL pig, have adapted to their homeland’s environmental conditions and can make better use of fibrous plant tissues as an energy source [25,26,27].

The main goal of this study was to provide new knowledge to the concept of modern pig production with a greater focus on animal welfare, while also integrating sustainable systems. We specifically aimed to understand how the microbiome dynamics in AL pigs respond to sex conditions and a late dietary transition based on legumes and agro-industrial by-products rich in fiber.

## 2. Materials and Methods

The procedures used in this study were approved by the Bioethical Committee for Animal Experimentation (ORBEA) of Évora University (process GD/38814/2020/P1).

### 2.1. Animals, Experiment Design, and Sampling

A total of 30 Alentejano (AL) male pigs were raised outdoors from approximately 40 kg to test the influence of castration and a high-fiber feed on performance, carcass traits, meat quality traits, and blood biochemistry. The pigs were fed between 1.6 and 4.2 kg per day (weekly adjustments) with a fattening commercial diet until slaughter, which occurred at approximately 160 kg LW, and they had free access to water. The growing conditions are explained in more detail in a previous study [2]. The pigs were divided into three groups (*n* = 10 for each group), namely: C, which included male pigs surgically castrated using anesthesia and analgesia; I, which included intact male pigs; IExp, which included intact male pigs that were fed an experimental isoproteic and isoenergetic diet, making use of locally produced legumes and agro-industrial by-products, during the period between 130 and 160 kg LW. The compositions of the fattening commercial diet and the experimental diet are reported in Supplementary Table S1. Briefly, diets were analyzed for dry matter (UE 500, Memmert, Schwabach, Germany), total ashes, crude protein (N × 6.25) (Kjeldatherm KB-20, Gerhardt, Bonn, Germany, and Kjeltec Auto 1030 Analyzer, Tecator, Bristol, UK), neutral and acid detergent fibers, total and insoluble fibers, and total sugars [28]. Cellulose and total starch were determined according to ISO-6865 [29] and ISO-6493 [30], respectively. Digestible energy of the diets was calculated according to Noblet, et al. [31]. The diet formulations are proprietary information. Fecal samples from the pigs of each group were collected into sterile tubes two weeks before slaughter and were stored at −80 °C until further analysis. Three animals (two from group C and one from group I) were excluded from the analysis for either not providing enough starting material or not meeting minimum quality control standards.

### 2.2. DNA Extraction and Sequencing

The total DNA was extracted from the fecal samples of the C (*n* = 8), I (*n* = 9) and IExp (*n* = 10) groups following the instructions from the QIAmp PowerFecal Pro DNA Kit (Qiagen, Hilden, Germany). The DNA concentration was determined using a NanoDrop 1000 spectrophotometer (NanoDrop Technologies, Wilmington, DE, USA).

The hypervariable V3-V4 region of the ribosomal 16S was amplified according to the KAPA HiFi HotStart PCR Kit instructions, using 0.3 μM of each of the specific primers, Bakt_341F 5′–CCTACGGGNGGCWGCAG-3′ (forward) and Bakt_805R 5′–GACTACHVGGGTATCTAATCC-3′ (reverse) [32,33], and 50 ng of the template DNA in a 25 μL reaction. The amplification cycles comprised a 3 min denaturation step at 95 °C, followed by 35 cycles at 98 °C for 20 s, 55 °C for 30 s, and 72 °C for 30 s, and a final extension step at 72 °C for 5 min. The resulting products were further reamplified in limited-cycle PCR reactions to add sequencing adapters and dual indexes to both ends of the amplified target region following Illumina^®^’s standard protocols [34]. No template controls were included in both amplification assays. The PCR products were then one-step purified and normalized using the SequalPrep 96-well plate kit (Thermo Fisher Scientific, Waltham, MA, USA), pooled, and paired-end sequenced at 2 × 300 bp in the Illumina MiSeq^®^ sequencer with the Miseq Reagent Kit v3, according to the manufacturer’s instructions (Illumina, San Diego, CA, USA) at Genoinseq (Cantanhede, Portugal).

### 2.3. Bioinformatic and Statistical Analysis

The raw reads were obtained from the Illumina MiSeq^®^ System in the FASTQ format after demultiplexing. QIIME2 version 2020.2.0 [35] was used to generate the amplicon sequence variants (ASV) and to perform the taxonomic identification. The tool, DADA2 [36], was used to remove noise from the data and to create ASVs. The selection and allocation of the taxonomic data for the ASVs was performed with the q2 feature-classifier plugin [37] using the SILVA ribosomal RNA gene database version 138 as reference [38]. ASVs not assigned to the *Bacteria* kingdom or assigned to the mitochondrial family were removed from the abundance tables, which were used for the alpha diversity estimations. The abundance table was further filtered to remove ASVs with a summed value lower than 100 reads in all samples. The table was then used for the composition and beta diversity analysis and to identify differentially abundant ASVs. Statistical analyses were executed using the R software version 4.3.0 [39] in RStudio version 2023.03.0 Build 386 [40]. Plots were generated with ggplot2 version 3.4.2 [41].

Alpha diversity estimations of richness (Observed and Chao-1) and diversity (Shannon and Simpson) were calculated with phyloseq version 1.44.0 [42]. The normality of the data was tested with the Shapiro test. The index values were compared using an analysis of variance (ANOVA) followed by Tukey’s test or the Kruskal-Wallis test, then followed by Dunn’s test with the Bonferroni correction.

Beta diversity was estimated with the Principal Coordinate Analysis (PCoA) using the Bray–Curtis dissimilarity in phyloseq. The differences in the microbiome composition between groups were determined with PERMANOVA, followed by a pairwise PERMANOVA using the adonis function in the vegan package version 2.6-4 [43] with 1000 permutations. The Benjamini–Hochberg procedure for multiple comparison corrections was used after testing for homoscedasticity with the betadisper function in the vegan package.

The Analysis of Compositions of Microbiomes with Bias Correction 2 (ANCOM-BC2) algorithm [44] version 2.2.0 was used to differentiate the abundance of taxa between the three groups at the phylum, family, and genus levels, tested for multiple comparison corrections with the Benjamini–Hochberg method. Results were considered statistically significant when the *p*-value was equal or less than 0.05. ANCOM-BC2 tested bias-corrected abundances for structural zeros (presence/absence between groups). The identified taxa were considered differentially abundant and were not further analyzed for statistical differences.

## 3. Results

### 3.1. Sequencing Metrics of the Bacterial Communities

A total of 2,174,301 reads (80,528 ± 19,957 average reads per sample) were obtained for the bacterial communities after sequencing. After quality filtering, a total of 469,638 high-quality reads remained (17,394 ± 3748 average reads per sample), and 5651 ASVs were obtained (Supplementary Table S2).

### 3.2. Alpha Diversity

**Figure 1:**
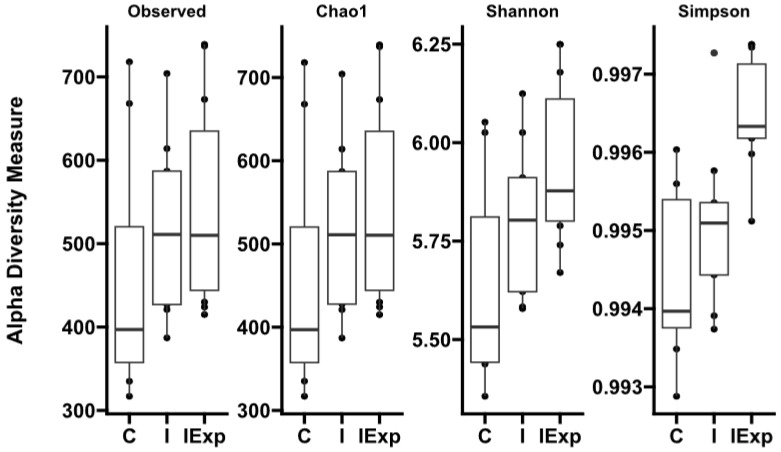
Alpha diversity indexes of the fecal bacterial communities of adult male Alentejano pigs according to the groups: C—Castrated, I—Intact, and IExp—Intact following an experimental diet. Indexes were calculated with phyloseq.

Alpha diversity indexes were estimated for richness (Observed and CHAO1) and diversity (Shannon and Simpson) (Figure 1).

No differences were observed between the groups for the richness indexes. However, the Shannon and Simpson indexes determined that the fecal bacterial communities differed between the C and the IExp groups (*p* < 0.05). Furthermore, the Simpson index also indicated a difference between the I and IExp groups (*p* < 0.05) (Table 1). The full list of alpha-diversity estimations per sample can be found in Supplementary Table S3.

### 3.3. Composition

**Figure 2:**
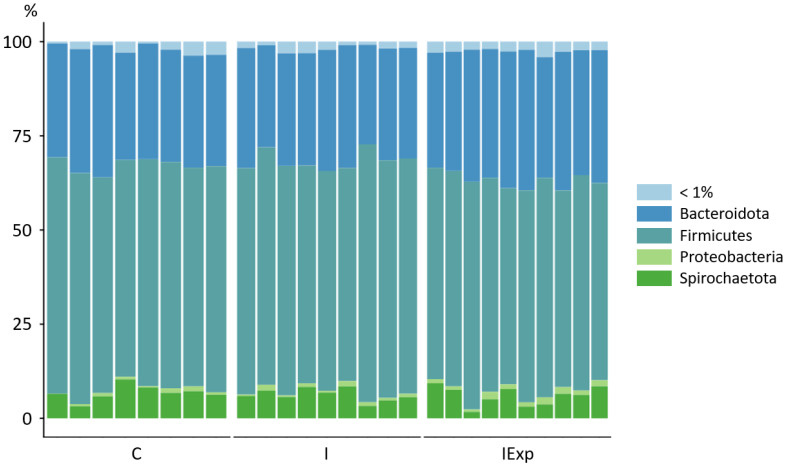
Representation of relative abundances of the fecal bacterial communities of Alentejano pigs at the phylum level according to the experimental groups. Groups: C—Castrated, I—Intact, and IExp—Intact following an experimental diet.

The bacterial communities were dominated by members of the Firmicutes phylum, with an average of 59% of the total abundance. Bacteroidota was the second most abundant phylum, with an average of 31.6% of the total abundance. The Spirochaetota phylum was the third most abundant, representing an average of 6.3% of the total abundance, while the phylum Proteobacteria represented ~1.0% (Figure 2).

Differences between the experimental groups were observed at lower taxonomical levels. *Lactobacillaceae* of the Firmicutes phylum was the most abundant family in both the C and I groups (24.7 and 24.0%, respectively). Meanwhile, *Prevotellaceae* of the Bacteroidota phylum was the most abundant family in the IExp group with 17.7% and the second most abundant family in the C and I groups (15.7 and 15.1%, respectively). At the genus level, *Lactobacillus* and *Treponema* were the two most abundant in both the C (24,7 and 6.6%, respectively) and I (23.9 and 6.0%, respectively) groups. In the IExp group, *Lactobacillus* was also the most abundant genus (14.8%), but the second was *Prevotella*, with a total of 6.6%. The full list of relative abundances for the three taxonomic levels of the bacterial communities can be found in Supplementary Table S4.

**Figure 3:**
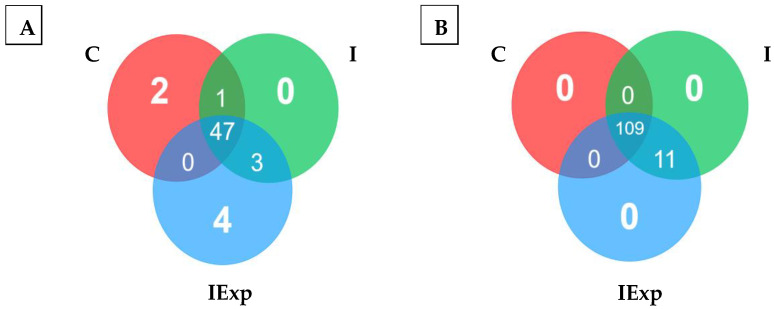
Venn diagram of ASVs at the family (**A**) and genus (**B**) levels according to the groups. Groups: C—Castrated, I—Intact, and IExp—Intact following an experimental diet.

Most of the taxonomic families identified were common to the three groups (47 families) (Figure 3A), with WPS-2, UCG-010, unclassified Bacteroidia, and *Monoglobaceae* found exclusively in the IExp group. Regarding the genera, most were also common to the three groups (109 genera) (Figure 3B), with 11 genera exclusively identified in the IExp group, namely: *Lachnospiraceae*, *Veillonella*, *Selenomonas*, *Bradymonadales*, *Lachnospiraceae* UCG-008, unclassified *Lactobacillales*, [Eubacterium] brachy group, *Lachnospiraceae* NK4B4 group, *Bacteria*, unclassified *Peptococcaceae,* and T34. The complete list of shared and specific taxa of the bacterial communities between the groups can be found in Supplementary Table S5.

### 3.4. Beta Diversity

**Figure 4:**
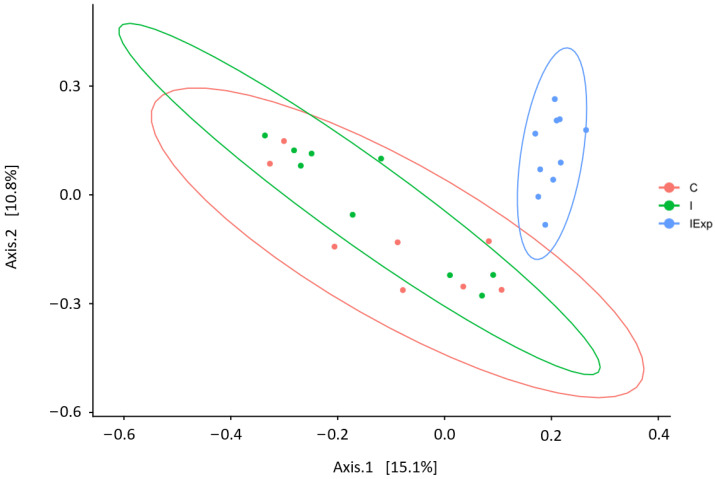
Principal Coordinates Analysis of the fecal bacterial communities of the three groups based on the Bray–Curtis dissimilarity. Ellipses were drawn at a 95% confidence level. Groups: C—Castrated, I—Intact, and IExp—Intact following an experimental diet.

The structure of the bacterial communities differed among the groups, according to the PCoA based on the Bray–Curtis dissimilarity (PERMANOVA: R2 = 0.167, *p* = 0.000999) (Figure 4). No statistically significant differences separated C and both of the intact groups (I and IExp); however, the IExp group differed from the C (pairwise PERMANOVA *p* < 0.002) and the I ones (pairwise PERMANOVA *p* < 0.002).

### 3.5. Differential Abundances and Identification of Structural Zeros

Bacterial communities present in the guts of the pigs were analyzed for differential abundance to identify taxa influenced by sex or the experimental diet using ANCOM-BC2. At the phyla level, Cyanobacteria, WPS-2, and an OTU only identified at the kingdom level—indicated as Bacteria—were almost absent from the three groups (detected as structural zeros). Patescibacteria were not detected in the C group, while Desulfobacterota and Elusimicrobiota were absent (structural zeros) from the C and I groups. The bias-corrected abundances of the other phyla did not change among the groups (Supplementary Table S6).

The *Ruminococcaceae* family was differentially abundant between groups I and IExp and between groups C and IExp (total abundances of 2.25% in group C, 2.81% in group I, and 5.77% in group IExp). No other observable abundance differences were detected in the remaining 36 families. Absence/presence patterns were detected in 20 families that contributed, each with less than 1% of the total abundance. The families *Gastranaerophilales*, *Bradymonadales*, and *Monoglobaceae*, an undescribed T34 family of the Proteobacteria phylum, an undescribed WCHB1-41 family of the Verrucomicrobiota phylum, an undescribed family of the WPS-2 phylum, and an OTU only classified at the Bacteria kingdom level were present in very low abundances in the three groups (Supplementary Table S7).

**Figure 5:**
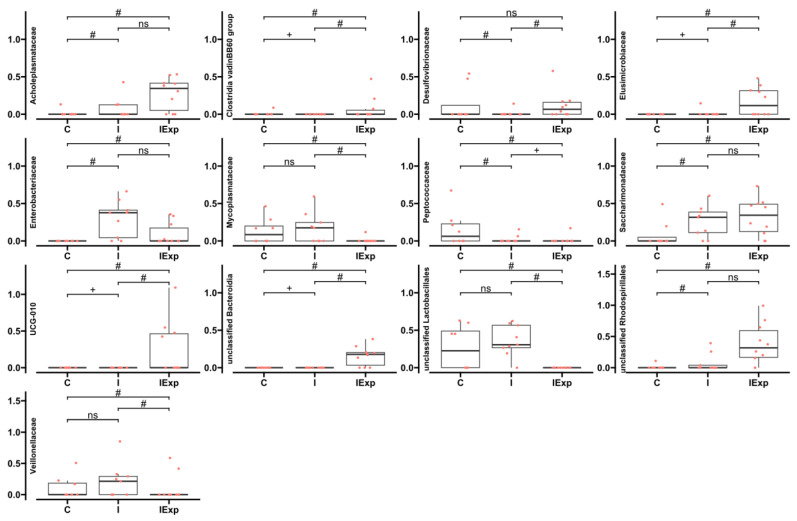
Differentially abundant families of fecal bacterial communities in pig groups analyzed with ANCOM-BC2. The boxplots represent the distribution of sample abundances in percentage by median, interquartile range, minimum and maximum values for the indicated families. +—both groups are absent in the family; #—family is differentially abundant due to structural zero in one of the groups; ns—family is not differentially abundant (*p* > 0.05). Groups: C—Castrated, I—Intact, and IExp—Intact following an experimental diet.

Four other presence/absence patterns were detected within the 13 remaining families. The families *Mycoplasmataceae* and *Veillonellaceae* and an unclassified *Lactobacillales* family were absent from the IExp group. *Desulfovibrionaceae* and unclassified Bacteroidia, *Elusimicrobiaceae* of the Elusomicrobiota phylum, and the Firmicutes families *Clostridia* vadinBB60 group and UCG-010 were only detected in group IExp. The families *Acholeplasmataceae*, *Saccharimonadaceae*, and *Enterobacteriaceae* and an unclassified *Rhodospirillales* were not observed in group C. Finally, *Peptococcaceae* was only detected in group C (Figure 5, Supplementary Table S7).

**Figure 6:**
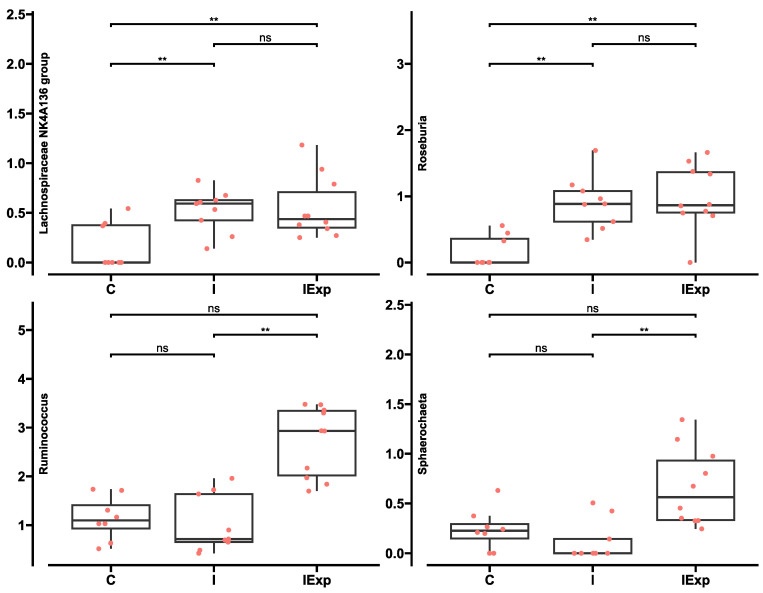
Differentially abundant genera of the fecal bacterial communities between pig groups analyzed with ANCOM-BC2. The boxplots represent the distribution of sample abundances in percentage by median, interquartile range, minimum, and maximum values for the indicated genera. ns—genus is not differentially abundant (*p* > 0.05), **—genus is differentially abundant between groups (*p* < 0.01). Pig groups: C—Castrated, I—Intact, and IExp—Intact following an experimental diet.

At the genus level, four genera were differentially abundant (q < 0.05) between groups, 48 genera displayed a presence/absence pattern, and 68 genera had similar abundances between the groups (Supplementary Table S8). Regarding the four genera detected as differentially abundant, *Ruminococcus* and *Sphaerochaeta* were significantly more abundant in the IExp group when compared to the C and I groups (q < 0.01), while *Lachnospiraceae* NK4A136 group and *Roseburia* were less abundant in the C group when compared to the I and IExp groups (q < 0.01) (Figure 6).

Eighteen genera were represented at very low abundances in all groups, *Gastranaerophilales*, *Bradymonadales*, [Eubacterium] xylanophilum group, [Eubacterium] brachy group, *Dorea*, *Lachnospira*, *Lachnospiraceae* NK4B4 group, *Lachnospiraceae* UCG-008, *Moryella*, *Monoglobus*, UCG-002, *Intestinibacter*, *Mitsuokella*, *Selenomonas*, an undescribed T34 family of the *Proteobacteria* phylum, an undescribed WCHB1-41 family of the Verrucomicrobiota phylum, an undescribed family of the WPS-2 phylum, and an OTU only classified at the Bacteria kingdom level (Supplementary Table S8).

Different presence/absence patterns were also detected at the genera level. *Megasphaera*, *Veillonella*, and *Holdemanella* were found to be significantly more abundant in the I group when compared to the C and IExp groups. On the other hand, UCG-004, *Clostridia* vadinBB60 group, [Eubacterium] ruminantium group, *Lachnospiraceae* UCG-007, *Paludicola,* UCG-010, an unclassified genus of Bacteroidia of the Bacteroidota phylum, *Desulfovibrio*, *Mailhella,* and *Elusimicrobium* were only observed in the IExp group. The genera *Mycoplasma*, [Ruminococcus] gauvreauii group, and *Agathobacter* and an unclassified *Lactobacillales* genus were absent from the IExp group. An unclassified *Peptococcaceae* genus and UCG-009 were only observed in group C. The genera *Anaeroplasma*, Candidatus Stoquefichus, *Anaerostipes*, *Marvinbryantia*, *Butyricicoccus*, *Ruminococcaceae* Incertae Sedis, *Subdoligranulum*, Candidatus Saccharimonas, and *Escherichia-Shigella* and an unclassified *Rhodospirillales* genus were absent from group C (Supplementary Table S8).

## 4. Discussion

Recent developments in next-generation sequencing have propelled research on the gut microbiome, expanding our understanding of complex host-microbiome interactions in pigs. Different studies have revealed how microbial communities participate in the breakdown and fermentation of dietary components and how maintaining a stable and diverse microbiome is essential to avoid digestive disorders and disease susceptibility [45,46,47].

In this study, we explored how the gut microbiome of intact AL pigs is influenced by surgical castration and a dietary modification based on locally produced legumes and agro-industrial by-products rich in fiber. The phenotypic characterization of the animals used in this study has been previously shown [2]. Briefly, intact pigs presented faster growth as well as leaner carcasses and were richer in unsaturated fatty acids when compared to castrated pigs. The higher lipid synthesis and deposition in the C pigs was later confirmed by a transcriptomics study [48]. The experimental diet did not have any significant negative impacts on growth, carcass, and meat quality traits of IExp when compared to I pigs, the latter consuming commercial diets. This fiber-rich diet also did not significantly reduce the amount of skatole quantified in the subcutaneous fat of those animals. However, both of the boar taint compounds tested, androstenone and skatole, were found to be below the consumers’ rejection threshold in all groups. These results suggest a positive influence of genetics and/or the rearing system. The potential for raising Alentejano pigs without castration, thus enhancing animal welfare, requires further validation through future research.

Our microbiome results indicate that the most abundant phylum present in the gut of all groups is Firmicutes (C—59.68, I—61.46, and IExp—55.87%), followed by Bacteroidota (C—30.57, I—29.77, and IExp—34.29%). Previous studies point out that these two phyla can account for over 90% of the total 16S sequences identified [13,49], and our results are identical to these findings. The decreased abundance of Firmicutes and increased abundance of Bacteroidota in the IExp group agree with previously described data, suggesting that diets with increased fiber content increase the abundance of Bacteroidota at the expense of Firmicutes [50]. This change can maximize energy absorption from dietary fiber and might have contributed to the IExp group presenting the highest average daily gain, particularly during the dietary transition period (130–160 kg; C—591.1, I—595.0, and IExp—732.2 g/day, *p* < 0.05) [2]. Meanwhile, the Firmicutes/Bacteroidota ratio has been proposed as a good obesity marker, particularly in humans and animals [51,52,53,54]. Firmicutes populations appear to be more efficient regarding energy production than Bacteroidota, resulting in a higher calorie intake and consequent weight gain. On the other hand, calorie-restricted diets tend to increase the abundance of Bacteroidota and reduce Firmicutes. However, the nature of the correlation between this ratio and body composition is still not clear, since it can be related to causality or be a consequence of fatness [52,54,55]. In our study, despite the slaughter weight of all pigs being similar (~160 kg), the fat cuts percentage was significantly higher in only C pigs (*p* < 0.0001), while the I and IExp groups showed similar values (C—28.7, I—25.0, and IExp—24.7%) [2]. Therefore, the observed lower distribution of Firmicutes and higher distribution of Bacteroidota in IExp should only be associated with the increase in dietary fiber. The fatness phenotype is suggested to be less influential in shaping these communities, at least at the phylum level.

*Ruminococcaceae* of the Firmicutes phylum was the only family with a statistically different abundance among the three experimental groups (q < 0.05), presenting significantly higher values in IExp pigs (C—2.25, I—2.81, and IExp—5.77%). The family is composed of strictly anaerobic bacteria, and its higher relative abundance is generally associated with better gut health and function [56,57]. This beneficial attribute is related to their ability to ferment complex carbohydrates into butyrate and other short-chain fatty acids, which is fundamental to provide energy for colonocytes, stimulate cell proliferation, and suppress pro-inflammatory cytokines [58,59]. Other studies have also identified a link between these microorganisms and meat quality and lipid metabolism improvements [60,61,62]. Furthermore, the suggested higher production of short-chain fatty acids in the IExp group may have positively influenced the average daily gain, as previously mentioned, as well as the lower feed conversion ratio observed (q < 0.01; C—4.63, I—4.21, and IExp—3.90 kg/kg) [2,59,60,63,64].

At the genus level, a total of four genera were found to be differentially abundant among the groups, namely *Ruminococcus*, *Sphaerochaeta*, *Roseburia* and *Lachnospiraceae* NK4A136 group. Both *Ruminococcus* (q < 0.01; C—1.13, I—1.11, IExp—2.66%) and *Sphaerochaeta* (q < 0.01; C—0.25, I—0.12, IExp—0.67%) presented significantly higher abundances in IExp when compared to I pigs. Members of the *Ruminococcus* genus are Gram-positive anaerobic bacteria that display a mutualistic relationship with their hosts and contribute to their overall gut health. In pigs, as with other mammals, *Ruminoccocus* spp. can ferment complex fiber polymers, particularly cellulose and hemicellulose, allowing the formation of short-chain fatty acids that provide the beneficial attributes previously described for Ruminococcaceae. The species identified in our data include *Ruminococcus callidus* and *Ruminococcus flavefaciens*. The first one is known for its polysaccharide degradation activity, such as starch and xylan (hemicellulose) [65], while the second can assemble a multi-enzymatic system that can degrade cellulose like most ruminococci [66,67]. Besides the higher content of total fiber in the experimental diet when compared with the commercial fattening diet (40.2 and 26.9 g/100 g dry matter, respectively), the higher abundance of these species agrees with the higher contents of cellulose (10.4 vs. 6.5 g/100 g DM) and hemicellulose (16.7 vs. 13.2 g/100 g DM). The experimental diet supplied promoted the growth of *Ruminococcus* spp. since they were provided with more substrates to metabolize.

The *Sphaerochaeta* genus includes anaerobic, mesophilic, and halophilic bacteria, and, as with *Ruminococcus* spp., their genomes comprise numerous carbohydrate fermentation genes [68]. An increased abundance of *Sphaerochaeta* spp. may indicate that the gut microbiota in IExp is actively fermenting more fiber-rich components that bypassed digestion in the small intestine due to the lack of enzymatic systems that can process such compounds. These are later transformed into short-chain fatty acids, such as butyrate, acetate, and propionate, and contribute to the overall gut health. *Sphaerochaeta* spp. have previously been shown to have a negative correlation with the average daily gain, backfat thickness, and slaughter weight in Enshi pigs [69]. However, our results do not support any of these hypotheses. The finishing body weight did not change between our groups (~160 kg), but the average daily gain was found to be significantly higher in IExp pigs (*p* < 0.05; C—591.1, I—595, and IExp—732 g/day), indicating a positive correlation. Backfat presented a significantly higher thickness for C pigs but no significant differences between I and IExp (C—68.2, I—47.8, and IExp—50.5 mm).

The genus *Roseburia* was found with a significantly lower relative abundance in castrated pigs when compared to both intact groups (q < 0.01; C—0.16, I—0.89, and IExp—1.00%). Five species integrate this genus of Gram-positive anaerobic bacteria that metabolizes dietary components like fiber and complex carbohydrates into short-chain fatty acids, mostly butyrate. *Roseburia* spp. have previously been proposed as a health marker. Several metabolic disturbances have been associated with lower abundances of these species, such as obesity, type 2 diabetes, and inflammatory bowel syndromes [59,70]. This agrees with our results [2] since C pigs presented significantly higher carcass fat cuts (including belly and backfat) (*p* < 0.01; C—28.7, I—25.0, and IExp—24.7%), backfat thickness (*p* < 0.01; C—68.2, I—47.8, and IExp—50.5 mm), and circulating levels of triacylglycerols (*p* < 0.01; C—0.53, I—0.37, and IExp—0.30 mmol/L) when compared to both intact groups.

Similar to *Roseburia*, the genus *Lachnospiraceae* NK4A136 group was found to be significantly lower in C pigs when compared to both intact groups (q < 0.01; C—0.19, I—0.55, and IExp—0.57%). This genus is also responsible for producing butyrate from fermented complex carbohydrates, contributing to better gut health [71]. The abundance of *Lachnospiraceae* NK4A136 group in mice fed with high-fat diets has previously been shown to be significantly lower when compared to a balanced diet and was negatively correlated with circulating triacylglycerol levels [72,73]. These results have suggested a probiotic effect of *Lachnospiraceae* NK4A136 group, which agree with our results since C pigs presented the fatter phenotype and the highest circulating triacylglycerol levels [2].

The relationship between male castration (removal of the testes) and gut microbiota is complex and still not clear. The production downfall of sex hormones in castrated pigs is known to play a decisive metabolic role, promoting adiposity and influencing body composition [74]. Interestingly, studies in mice have determined that castrated obese-prone genotypes only develop their phenotype, even at high-fat diets, when certain microbiota is present in the gut [75]. Even though no such studies exist in pigs, the relevance of the multiple interactions of the microbiota–sex–diet axis in forging a determined phenotype is certain.

Higher relative abundances of *Lactobacillus* spp. have previously been associated with lower fiber digestibility pig groups [49], a result that considerably agrees with our results. *Lactobacillus* was ~38% less abundant in IExp when compared to the I and C groups (C—24.67, I—23.94, and IExp—14.80%), although not attaining statistical significance. This is probably due to the statistical approach implemented being focused on minimizing the occurrence of false positives. Other examples of interesting results that did not attain statistical significance include the families *Lachnospiraceae* (C—11.97, I—15.04, and IExp—15.88%) and *Prevotellaceae* (C—15.69, I—15.13, and IExp—17.68%) and the genus *Streptococcus* (C—2.02, I—0.38, and IExp—0.12%). Both families, *Lachnospiraceae* and *Prevotellaceae,* include anaerobic species that can ferment dietary fiber components and produce short-chain fatty acids, being generally considered important families of a healthy and stable gut microbiota [76,77]. Surgical castration appears to have affected the proportion of *Lachnospiraceae* suggesting lower gut health, probably associated with the fatter phenotype of these pigs, while the fiber-rich diet provided more substrates for *Prevotellaceae*, promoting its higher abundance. The *Streptococcus* genus encompasses Gram-positive bacteria that integrate the commensal microbiota of the gut of pigs. Higher abundances of *Streptococcus* spp. have recently been associated with circulating biomarkers of systemic inflammation and a response to infection in humans [78]. The same study proposed a link between the abundance of *Streptococcus* spp. and the development of coronary atherosclerosis. The two species identified include *Streptococcus hyointestinalis* and *Streptococcus macedonicus*. *S. hyointestinalis* was first isolated in the intestine of pigs [79] and has shown potential in producing anti-pathogen compounds similar to bacteriocin [80]. *S. macedonicus*, on the other hand, is known to participate in the fermentation of dairy and dairy-derived products. Certain strains of this species can also produce exopolysaccharides and bacteriocins that exhibit anti-clostridium activity [81].

From the genera that displayed presence/absence patterns, it should be highlighted that *Desulfovibrio*, *Elusimicrobium*, *Lachnospiraceae* UCG-007, and *Paludicola* were identified as structural zeros in groups C and I but presented a relevant abundance in IExp. These microorganisms have diverse metabolic activities, such as fermentation of complex carbohydrates, sulfate reduction, and other anaerobic processes that can have significant impacts on the overall gut condition and function [82,83,84,85]. Furthermore, several genera were absent from the C animals and were relevant in both intact groups, such as the cellulose/methylcellulose-degrading *Marvinbryantia* [86] and two butyrate-producing genera, namely *Butyricicoccus* [87] and *Subdoligranulum* [88]. These last two are potential healthy biomarker genera that frequently present lower abundances in obese individuals [89,90]. Finally, the presence of *Mycoplasma* in both the C and I groups and its absence in IExp could indicate a potential systemic infection [91]. Nevertheless, not all *Mycoplasma* species are pathogenic, and their presence in the C and I pigs may only suggest a disruption to the microbial balance of the gut. IExp displayed a more stable and healthy microbial community.

The alpha diversity indexes measuring species richness (observed and Chao1) did not statistically differ among the groups, though it was numerically the lowest in the C pigs and highest in the IExp pigs. Regarding the indexes that take into account the evenness of the species distribution in addition to the number of species, both Shannon and Simpson indicated significantly higher diversity within IExp when compared to the C pigs. Nevertheless, while the Shannon index presented intermediate values in the I pigs (not different to either the C or IExp), the Simpson index identified IExp as the group with the highest diversity compared to both the C and I groups. The divergence in composition among groups is particularly clear in the PCoA performed to test the beta diversity (diversity between samples), where the C and I pigs were clustered together and IExp remained isolated.

These results suggest that IExp presented a gut microbial community more diverse and with more species, especially contrasting with the C pigs. The experimental diet shifted the bacterial composition present in the gut to potentiate distinct functional capabilities to address the new requirements imposed by the dietary transition. Higher biological diversity is also indicative of a more stable and resilient community, less susceptible to environmental disturbances, and capable of establishing better symbiotic interactions with its host, contributing to homeostasis [92].

The different gut microbial composition of the host will dictate its ability to process food and obtain energy and nutrients, which will influence the quantity and quality traits of the meat and meat products obtained [47]. However, different bacteria will process specific substrates, and the production of the associated short-chain fatty acids is also substrate-dependent [93]. Fiber-rich diets induce multivariate effects on the gut microbiota of pigs with boar taint. These include changes in diversity, competition among microbial species, and adjustments in metabolic pathways. New carbon-based substrates can shift the microbial composition from protein to hydrocarbon-processing bacteria, leading to a decline in the synthesis and accumulation of skatole [94].

Our results did not identify a differentially abundant genus associated with boar taint, which may be related to the fact that, as mentioned before, androstenone and skatole levels were low, below the consumers’ rejection threshold in all groups. The average skatole levels in all animals were lower than 4 ng/g and did not significantly differ among the groups, suggesting a positive effect of genetics and/or the rearing system [2]. Nevertheless, there are some genera within our results that can participate in the metabolism of L-tryptophan or its derivatives, which should be highlighted. Tryptophan can be converted to skatole in a four-step reaction that includes deamination, decarboxylation, dehydrogenation, and a last decarboxylation. The list of bacteria known to be involved in these processes include *Escherichia coli*, *Enterobacter cloacae*, *Clostridium* spp., *Lactobacillus* spp., *Pseudomonas* spp., *Bacteroides* spp., and *Olsenella uli* [95,96,97,98]. *Escherichia coli* and the genera *Enterobacter* and *Pseudomonas* were not identified in all samples. *Lactobacillus* was ~38% less abundant in IExp when compared to the I and C groups (C—24.67, I—23.94, and IExp—14.80%) as mentioned before, with a similar pattern to what was observed in the only *Clostridium* genus identified. *Clostridium sensu stricto 1* was found to be over 21% lower in the IExp (C—1.41, I—1.41, and IExp—1.14%), while the genus *Bacteroides* presented numerically higher values in the IExp when compared to the other groups (C—0.25, I—0.32, and IExp—0.89%). Finally, only eight reads of the OTU associated with the genus *Olsenella* (unidentified species) were assigned to one of the animals of the I group.

Diets enriched with fiber still hold promise as a strategy to manage the occurrence of boar taint in uncastrated male animals, despite the results in our study, suggesting that the genetic component of AL pigs in minimizing its occurrence is fundamental. Implementing such diets by making use of locally produced legumes and agricultural by-products can also prove useful in addressing broader goals of animals’ health and welfare and sustainable pig production.

## 5. Conclusions

To the best of our knowledge, this is the first microbiome study in Alentejano pigs. Here, we explored how the gut microbiome of intact AL pigs raised until ~160 kg is influenced by surgical castration and a dietary modification based on legumes and agro-industrial by-products rich in fiber. The experimental diet significantly increased the abundance of *Ruminococcus* spp. and *Sphaerochaeta* spp. (q < 0.05), which might have increased the concentration of short-chain fatty acids produced in the gut, such as butyrate. These might have contributed to the IExp group presenting a higher average daily gain, feed efficiency, and improved gut health. On the other hand, C pigs presented a significantly lower abundance of the potential probiotics, *Roseburia* spp. and *Lachnospiraceae* NK4A136 group (q < 0.01), species commonly associated with better gut health and individuals with a lower body fat composition. IExp pigs presented a gut microbial community that was more diverse and with more species when compared to the I and C groups. Furthermore, the experimental diet shifted the bacterial composition to potentiate distinct functional capabilities and address the new requirements invoked by the dietary transition. The genetic component or the rearing system applied are suggested to be the main impulse for the under-accumulation of boar taint compounds in AL pigs. Further studies are required to confirm this hypothesis. The gut microbiota is a dynamic ecosystem influenced by numerous factors, and understanding the complex relations involved is essential to advance the knowledge of microbe–host interactions and their implications for pig production systems. Diets enriched with fiber still hold promise as a strategy to manage the occurrence of boar taint in uncastrated male animals, and if sourced from locally produced legumes and agricultural by-products, they can also prove useful in addressing broader sustainability goals.

## Figures and Tables

**Table 1:** Alpha diversity indexes of the bacterial communities present in the fecal material of Alentejano pigs.

Indexes\Groups	C	I	IExp
Observed	458.5 ± 143.3	514.1 ± 97.9	544.5 ± 119.3
Chao1	458.5 ± 143.2	514.2 ± 97.8	544.8 ± 119.3
Shannon	5.640 ± 0.254 ^a^	5.791 ± 0.189 ^a,b^	5.938 ± 0.201 ^b^
Simpson	0.9943 ± 0.0010 ^a^	0.9959 ± 0.0010 ^a^	0.9964 ± 0.0007 ^b^

Notes: Statistically significant differences between two averages (*p* < 0.05) are indicated by a different superscript letter within each index. Groups: C—Castrated, I—Intact, and IExp—Intact following an experimental diet. Data is presented as value ± estimated standard deviation.

## Data Availability

Data will not be shared due to privacy restrictions.

## Supplementary Materials

The following supporting information can be downloaded at: https://www.mdpi.com/article/10.3390/vetsci10110641/s1, Table S1: Composition of the growing and experimental diets; Table S2: ASVs abundance table; Table S3: Alpha diversity indexes per sample; Table S4: Relative abundances (%) of microbial communities of the pig groups; Table S5: Composition of shared and specific taxa of the bacterial communities of the C, I and IExp pig groups; Table S6: Differential abundances of the pig groups analyzed by ANCOM-BC2 at phyla level; Table S7: Differential abundances of the pig groups analyzed by ANCOM-BC2 at family level; Table S8: Differential abundances of the pig groups analyzed by ANCOM-BC2 at genus level.
